# Mitochondrial DNA and genomic DNA ratio in embryo culture medium is not a reliable predictor for *in vitro* fertilization outcome

**DOI:** 10.1038/s41598-019-41801-1

**Published:** 2019-03-29

**Authors:** Xinyue Zhang, Yue Sun, Xin Dong, Jianming Zhou, Fubo Sun, Tingting Han, Ping Lei, Rurong Mao, Xuzhou Guo, Qi Wang, Penghao Li, Ting Qu, Jihua Huang, Lingxiao Li, Tianhua Huang, Ying Zhong, Jiang Gu

**Affiliations:** 1 Jinxin Research Institute for Reproductive Medicine and Genetics, Chengdu Jinjiang Hospital for Maternal and Child Health Care, 66 Jingxiu Road, Chengdu, 610066 China; 2 Department of Clinical Research, Yikon Genomics Co. Ltd., Building 26, 1698 Wangyuan Road, Fengxian District, Shanghai, 201499 China; 3 Laboratory of Molecular Pathology, Center of Molecular Diagnosis and Personalized Medicine, Provincial Key Laboratory of Infectious Diseases and Molecular Pathology, Shantou University Medical College, Shantou, China; 4 Department of Pathology, Beijing University Health Science Center, Beijing, China

## Abstract

To investigate the ratio of mitochondrial DNA to genomic DNA (mt/gDNA) in embryo culture medium as a possible predictor for embryonic development and pregnancy outcome, we collected a total of 93 embryo biopsy specimens from 52 women at the corresponding Day 3 (D3) and Day 5 (D5) embryo culture medium of *in vitro* fertilization. With the multiple annealing and looping-based amplification cycles method of next-generation sequencing for whole genome amplification, we examined the karyotype of the biopsy samples and the mt/gDNA ratio in the culture medium. Results showed that the ratio of mt/gDNA had an upward trend with decreasing trophectoderm levels with no significant difference. At the same time, from D3 to D5, the mt/gDNA ratio in the medium of embryos that failed to become blastocysts showed an upward trend, and the mt/gDNA ratio of medium from embryos that reached blastulation with successful pregnancy showed a decreasing trend, but the differences were not statistically significant. We conclude that there is a certain correlation between mt/gDNA ratio and early embryonic development, but it does not reach a level that can be used as a clinical predictor.

## Introduction

*In vitro* fertilization technology has become an important choice for many infertile couples as an effective treatment^1^. Assisted reproductive techniques (ART) account for the birth of more than 3.5 millions babies worldwide, and the number of IVF cycles performed increases every year^1–5^. However, the current average success rate is only about 20–30%^1,2,5^. Therefore, improving the success rate of embryo implantation has became a huge challenge for assisted reproductive technology.

Previous studies have shown that as women age (especially >35 years), the probability of aneuploidy appears to increase significantly^6,7^, contributing to infertility. The widely used embryo preimplantation genetic screening (PGS) technology can be broadly divided into nucleic acid detection and embryo imaging technique. The former is a traumatic procedure requiring cell biopsy that increases the risk of embryonic damage^8^. The latter is difficult to identify certain genetic defects that may not affect initial embryonic morphology but may seriously affect embryonic growth and development at later stages^9^. Therefore, there is an urgent need for a technology capable of non-invasively and rapidly assessing the genetic quality of embryos in order to improve the success rate of IVF.

Mitochondria are organelles that have an independent cell membrane within the eukaryotic cells and play an important role in cell survival^10,11^. Unlike other organelles, it has an independent DNA structure (mtDNA) that cords genetic information related to energy production and cellular metabolism^12,13^. Mitochondria and mtDNA also have a crucial impact on the early development of embryos^14–16^. Recent studies have found that when embryos entered the blastocyst stage, intracellular mitochondria multiplied significantly, and there were millions of mtDNA copies in a single trophoblast cell^17,18^. From blastocyst fluid and embryonic body DNA was released into the culture medium that can be extracted for measurement^19–21^. Ravichandran *et al*. suggested that the mtDNA/gDNA ratio in the embryo culture medium was related to embryonic development, and it could be used, instead of biopsied embryo cells, to predict the quality and the outcome of embryo for implantation^22^. Many attempts have been made to address this issue, Stigliani *et al*. and Hashimoto *et al*. believed that the mt/gDNA ratio of embryo culture medium was related to blastulation and was expected to have a predictive value, while Victor *et al*. believed that mtDNA content had no correlation with blastocyst ploidy, age or viability^23–25^. This controversy has not been solved up to now. Therefore we performed a study to address this controversy.

## Results

### The experimental group and the control group

**Figure 1:**
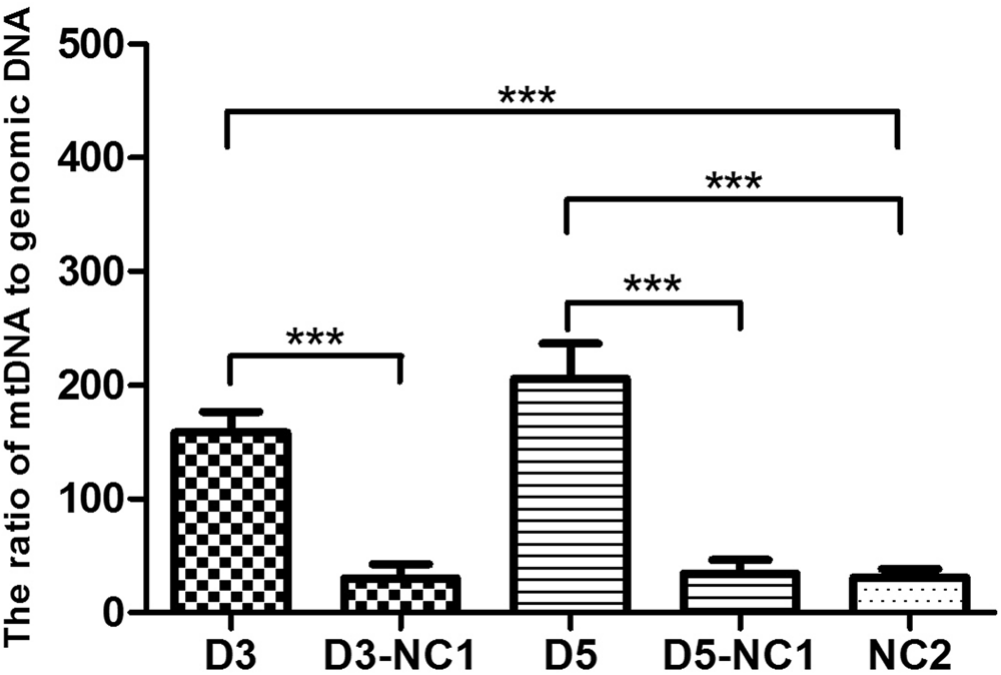
The ratio of mtDNA/gDNA in culture medium (n = 93) and negative control medium (n = 30). D3 and D5 were experimental groups, and D3-NC1 (n = 10) and D5-NC1 (n = 10) were the control groups that identically processed but did not contain embryo, NC2 (n = 10) is the unused culture medium. Data are means + SEM. ***P < 0.005.

We designed two kinds of controls, one was named NC1 that was culture medium underwent the corresponding culturing process but did not contain embryos, and the other was named NC2 that was not used for culturing. The average numbers of mt/gDNA ratios of the D3 group and the D5 group were 158.38 (SEM = 18.39) and 206.24 (SEM = 30.24), respectively, and there was no statistical difference (P = 0.09). The average numbers of mt/gDNA ratios of the D3-NC1 group, the D5-NC1 group and the NC2 group were 30.3 (SEM = 12.38), 34.2 (SEM = 12.27) and 31.10 (SEM = 7.64), respectively. The experimental results showed that the mt/gDNA ratio in the experimental groups (D3 and D5) are much higher than that of the corresponding control groups (D3-NC1 and D5-NC1) (P < 0.001). At the same time, there was also a significant difference between the experimental group and NC2 (P < 0.001), while there was no significant difference between NC2 and NC1 (D3-NC1 and D5-NC1) (Fig. 1).

### Embryonic chromosome euploidy and mt/gDNA ratio

**Figure 2:**
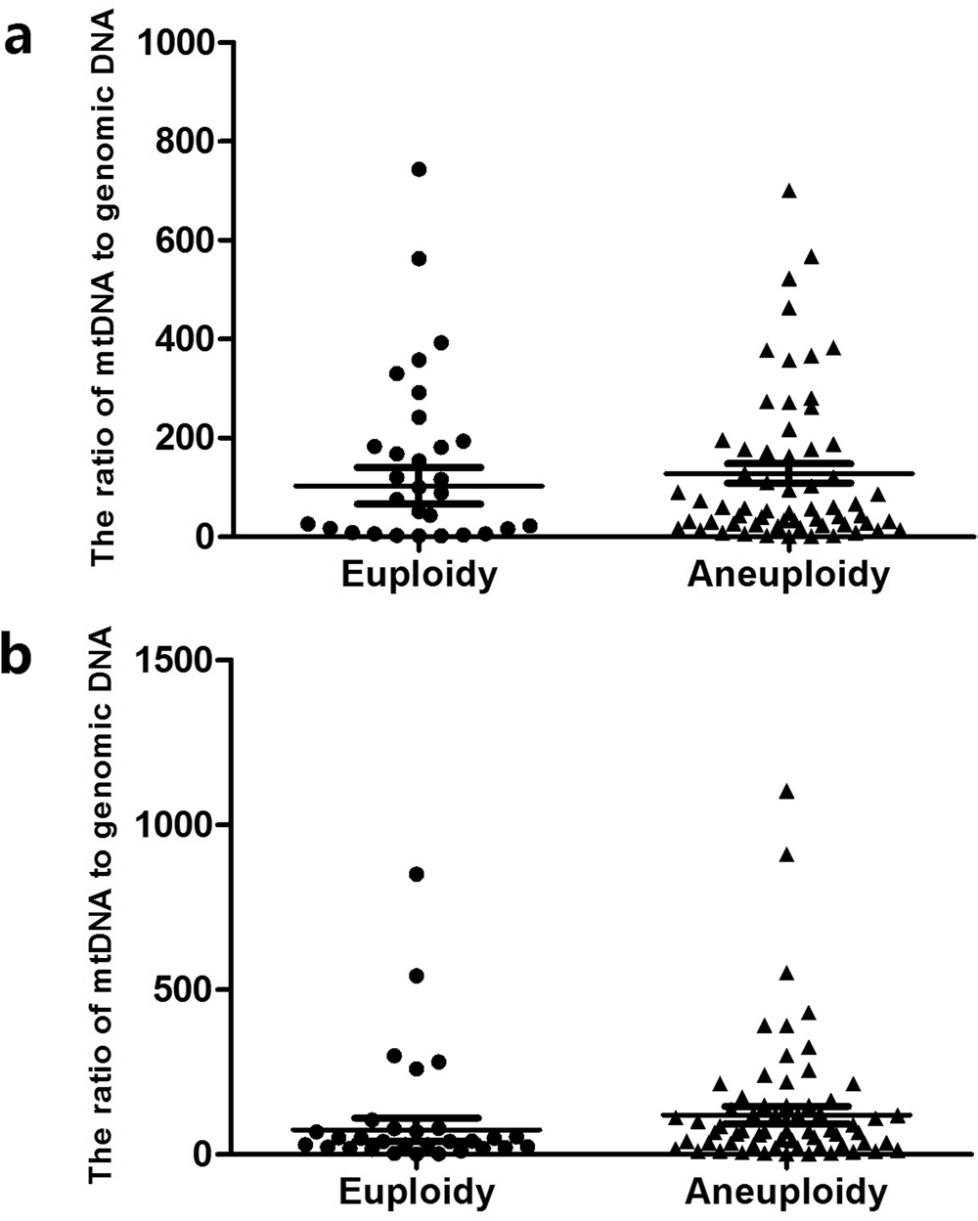
Corresponding mt/gDNA ratio in embryo culture medium when embryonic euploidy is different. At D3, the mt/gDNA ratio corresponding to different euploidy in the embryo culture medium (**a**). Mt/gDNA ratio corresponding to different euploidy in D5 embryo culture medium (**b**). We divided the samples into euploidy and aneuploidy based on chromosome ploidy of the embryo biopsy samples, as shown on the abscissa. The ordinate represents the ratio of mt/gDNA in the culture medium. One black dot or black triangle represents one sample. Data are means ± SEM.

To investigate the relationship between mt/gDNA ratio and embryo chromosomal ploidy in culture medium, we compared the mt/gDNA ratio of the D3 and D5 embryos in 93 embryos and chromosome ploidy of the corresponding biopsy samples. The embryo is usually biopsied at D5 if the embryo was developed into the Grade 4 blastocyst or later stage. If on D5 the embryo had not matured to the Grade 4 blastocyst, we continued to culture it to D6 and then performed the biopsy. The embryos after biopsy were vitrified. The number of eggs biopsied can be found in Supplement Table 1. The results showed that 30 (32.2%) embryonic biopsy samples had a normal chromosomal euploidy, and 63 cases (67.7%) had uneuploidy. In D3 culture medium, the average numbers of mt/gDNA ratios of the euploid group and the aneuploid group were 78.95 (SEM = 35.19) and 124.9 (SEM = 20.57), respectively, and there was no statistical difference (P = 0.087). In D5 culture medium, the average numbers of mt/gDNA ratios of the aneuploid group and the abnormal group were 37.52 (SEM = 33.40) and 107 (SEM = 25.45), respectively, and there was no statistical difference neither (P = 0.21) (Fig. 2).

### Blastocyst quality and mt/gDNA ratio

**Figure 3:**
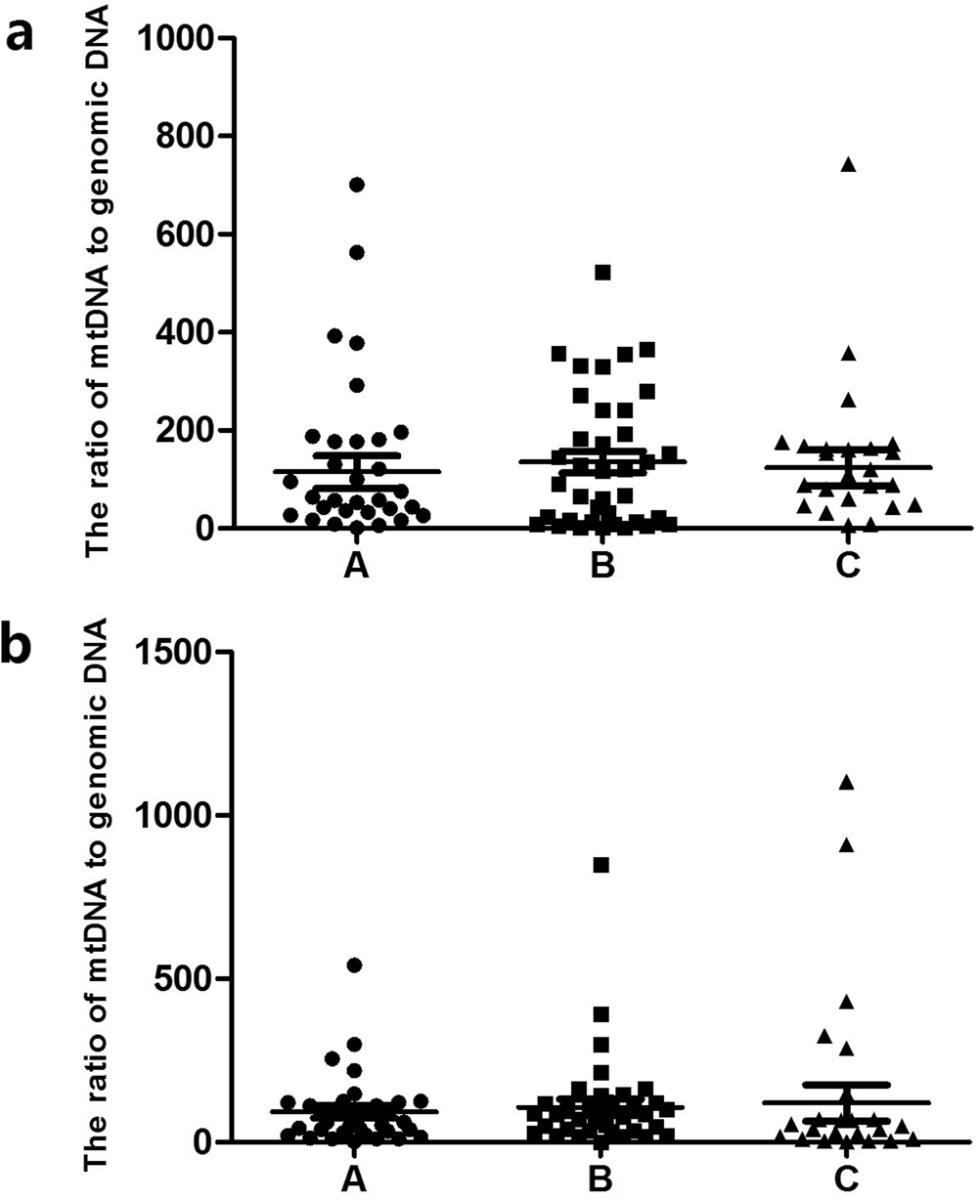
Corresponding mt/gDNA ratio in embryo culture medium when the score of ICM is different. At D3, the mt/gDNA ratio corresponding to different score of ICM in the embryo culture medium (**a**). Mt/gDNA ratio corresponding to different score of ICM in D5 embryo culture medium (**b**). According to the scores of ICM in each blastocyst (A–C), the samples were divided into three groups, A, B, and C, such as the abscissa. The ordinate represents the ratio of mt/gDNA in the culture medium. One black dot, black triangle or black square represents one sample. Data are means ± SEM.
  There were 33 TE Class A blastocysts (22 on Day 5 and 11 on Day 6), 38 TE Class B blastocysts (18 on Day 5 and 20 on Day 6) and 22 TE C-grade blastocysts (5 on day 5 and 17 on day 6). The results showed that the average numbers of mt/gDNA in the D3 medium, with the TE scores of A, B and C, were 62 (SEM = 16.62), 144.25 (SEM = 24.35) and 159.56 (SEM = 42.24), respectively, and there was no significant difference (P_AB_ = 0.45, P_BC_ = 0.48, P_AC_ = 0.42). At the same time, with the TE scores A, B and C, the average numbers of mt/gDNA in D5 medium were 29.25 (SEM = 14.19), 74.61 (SEM = 29.97) and 86.2 (SEM = 55.31), respectively with no significant difference (P_AB_ = 0.36, P_BC_ = 0.57, P_AC_ = 0.35). Interestingly, although there was no significant difference between the two on D3 and D5, they all increased with the decrease of TE series (Fig. 4).

1. We classified embryos based on inner cell mass (ICM) and TE morphology. There were 31 ICM Class A blastocysts (22 on day 5 and 9 on day 6), 38 ICM class B blastocysts (18 on day 5 and 20 on day 6) and 24 ICM class C (5 on day 5 and 19 on day 6).
  The results showed that the average numbers of mt/gDNA in the D3 medium, with the ICM scores of A, B and C, were 115.24 (SEM = 32.75), 137 (SEM = 22.13), and 122.81 (SEM = 36.75), respectively. There was no significant difference among them (P_AB_ = 0.64, P_BC_ = 0.93, P_AC_ = 0.70). At the same time, with the ICM scores A, B and C, the average numbers of mt/gDNA in D5 medium were 77.76 (SEM = 22.76), 84.9 (SEM = 33.28) and 93.7 (SEM = 68.54), respectively with no significant difference among them (P_AB_ = 0.36, P_BC_ = 0.19, P_AC_ = 0.053) (Fig. 3).

**Figure 4:**
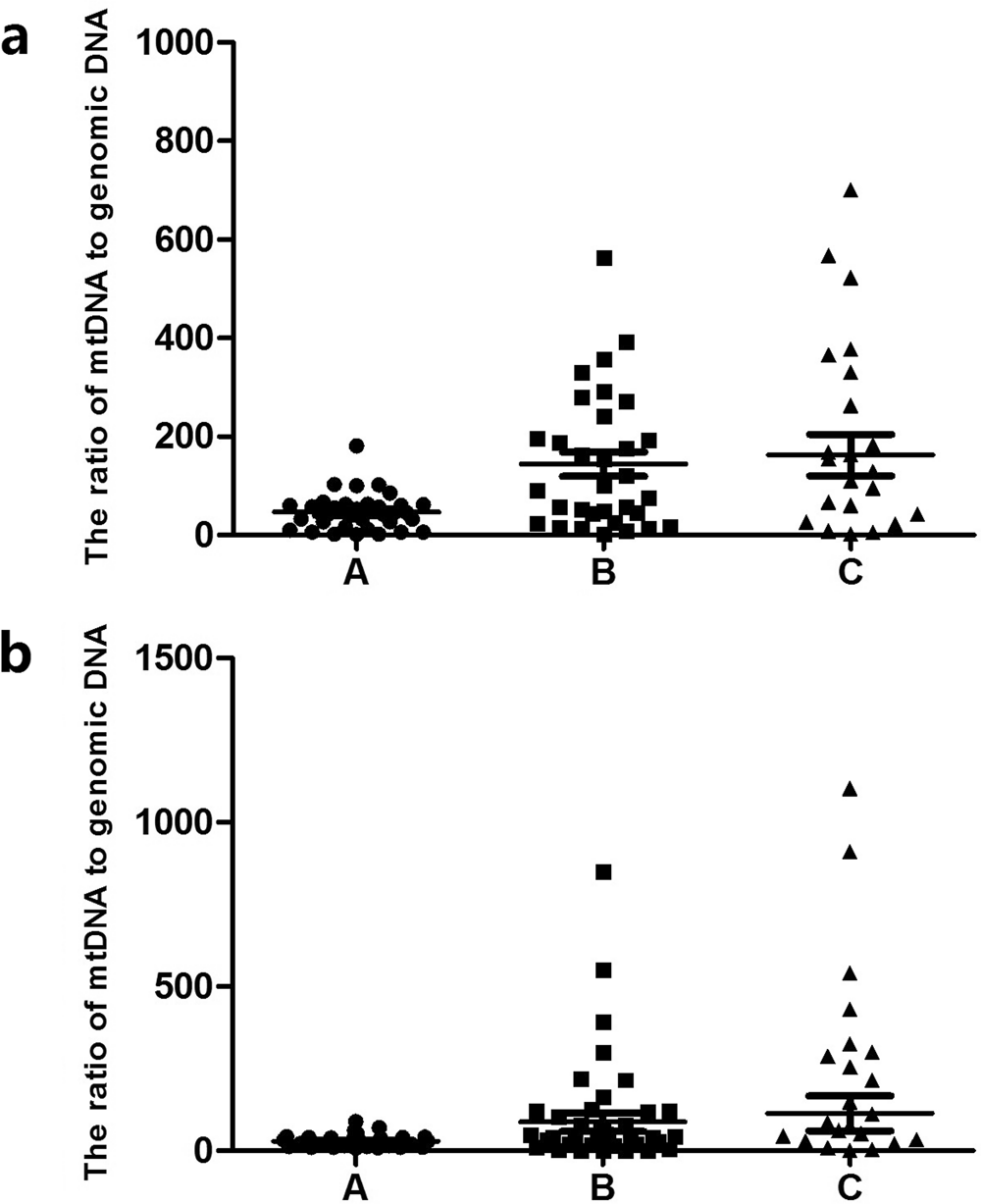
Corresponding mt/gDNA ratio in embryo culture medium when the score of TE is different. At D3, the mt/gDNA ratio corresponding to different score of TE in the embryo culture medium (**a**). Mt/gDNA ratio corresponding to different score of TE in D5 embryo culture medium (**b**). According to the scores of TE of each blastocyst (A–C), samples were divided into three groups, A, B, and C, such as the abscissa. The ordinate of the ordinate represents the ratio of mt/gDNA in the culture medium. One black dot, black triangle or black square represents one sample. Data are means ± SEM.
2. According to the proportion of fragments, the embryos were divided into three categories: Mild (0%), moderate (1–10%) and severe (>11%). The results showed that with the increase of the proportion of embryonic fragments from mild to moderate to severe, the average numbers of mt/gDNA in D3 medium were 134.48 (SEM = 21.57), 145.39 (SEM = 32.35) and 196.6 (SEM = 62.39), respectively, and there was no significant difference among them (P_AB_ = 0.36, P_BC_ = 0.65, P_AC_ = 0.49). At the same time, with the increase of the proportion of embryonic fragments from mild to moderate to severe, the average numbers of mt/gDNA in D5 medium were 85.81 (SEM = 20.95), 85.28 (SEM = 30.63) and 117.84 (SEM = 87.02), respectively, and there was no significant difference (P_AB_ = 0.90, P_BC_ = 0.34, P_AC_ = 0.40) (Fig. 5).

**Figure 5:**
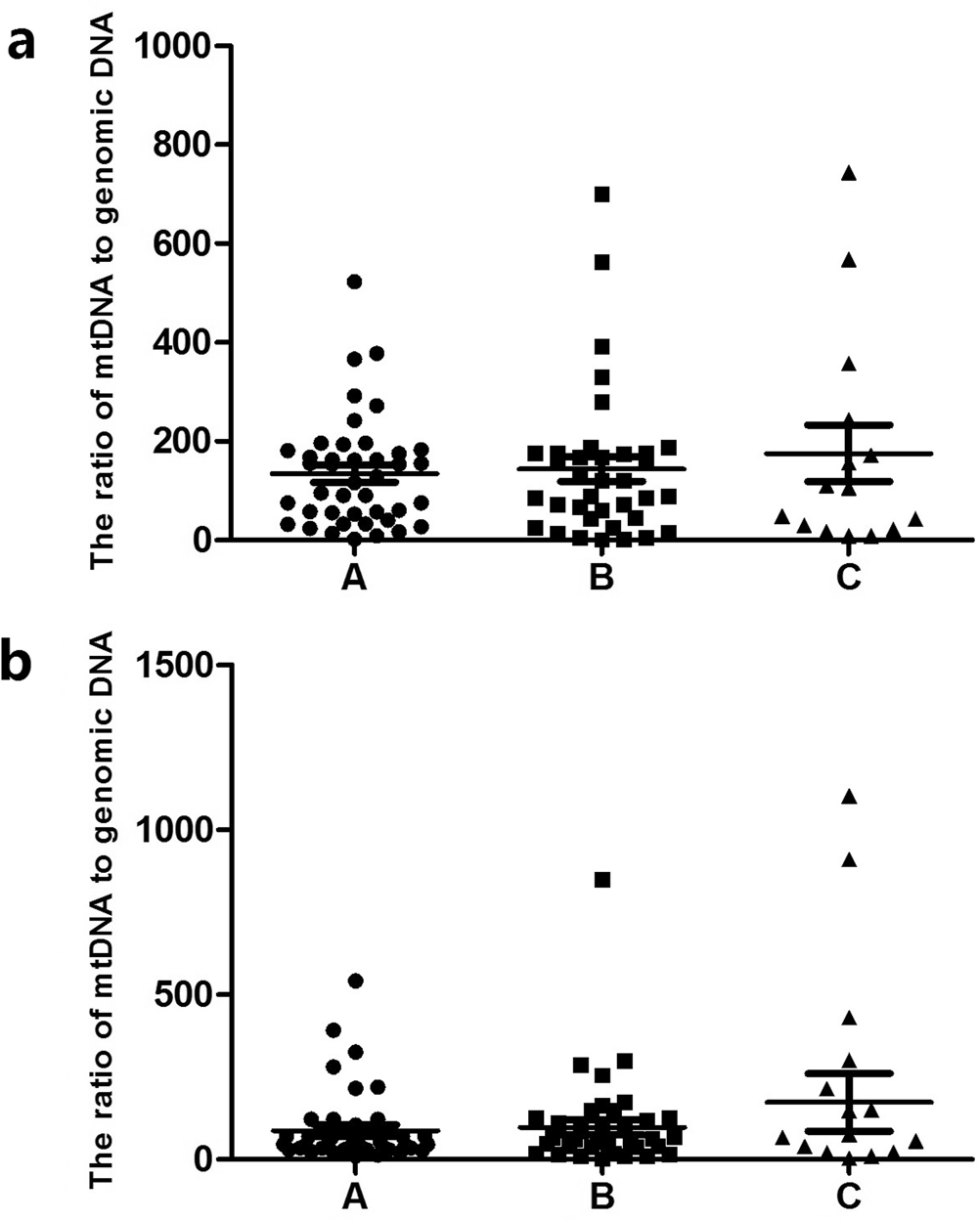
Corresponding mt/gDNA ratio in embryo culture medium when the proportion of embryo fragments is different. At D3, the mt/gDNA ratio corresponding to different proportion of embryo fragments in the embryo culture medium (**a**). Mt/gDNA ratio corresponding to different score of proportion of embryo fragments in D5 embryo culture medium (**b**). According to the scores of TE of each blastocyst (A–C), the samples were divided into three groups, A, B, and C, such as the abscissa. The ordinate of the ordinate represents the ratio of mt/gDNA in the culture solution. One black dot or black triangle or black square represents one sample. Data are means ± SEM.
3. Blastulation: The average numbers of mt/gDNA ratio between embryos developing to blastocyst stage (85/93) and embryos developing to blastocyst stage (Grade 4) failed (8/93) on D3 were 141.43 (SEM = 17.76) and 89.43 (SEM = 56.24) respectively without significant difference (P = 0.054), and 83.39 (SEM = 19.63) and 101.57 (SEM = 80.67) on D5 respectively without significant difference (P = 0.61) (Fig. 6).

**Figure 6:**
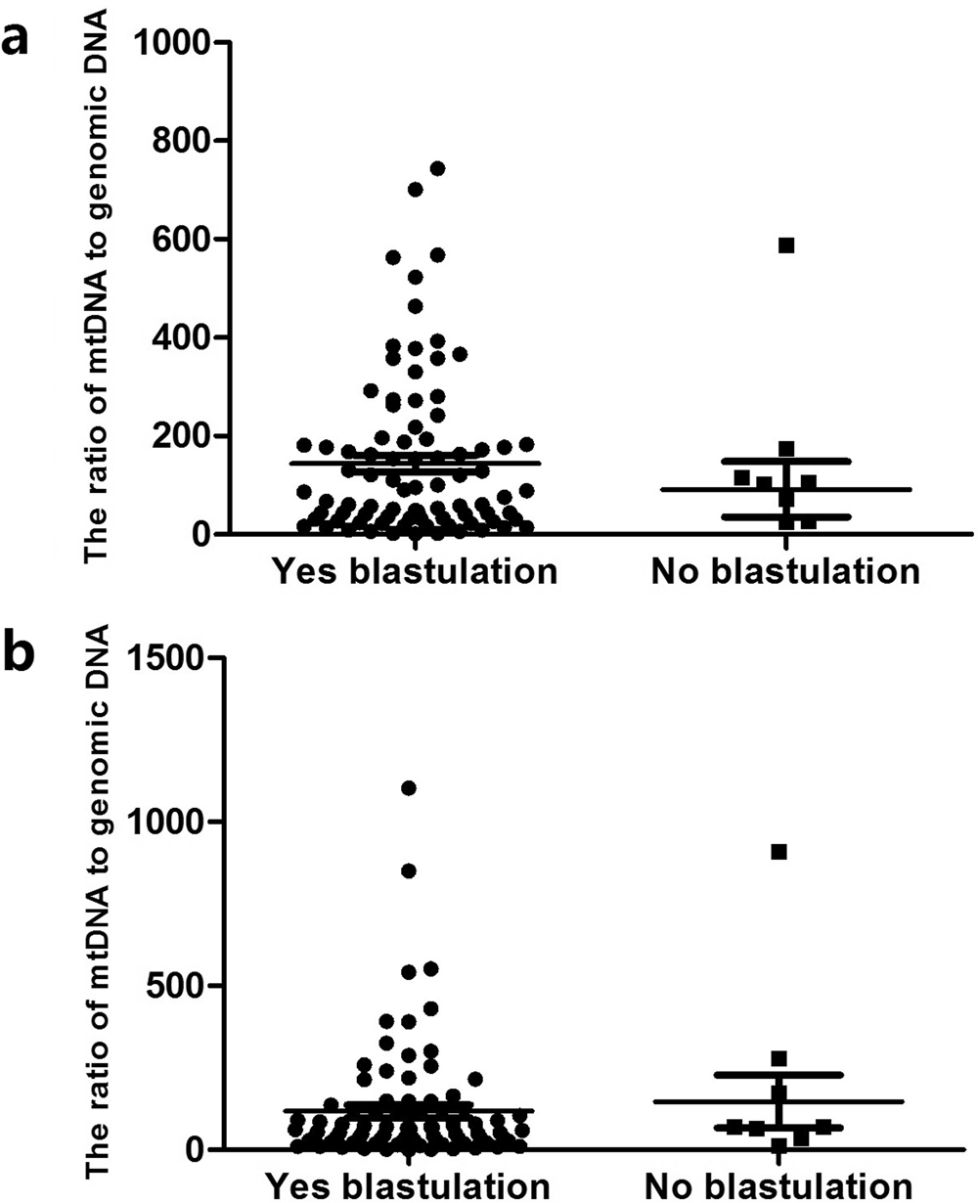
Mt/gDNA ratio in embryo culture medium corresponding to different outcome of blastulation. At D3, the mt/gDNA ratio corresponding to different outcome of blastulation in the embryo culture medium (**a**). Mt/gDNA ratio corresponding to different outcome of blastulation in D5 embryo culture medium (**b**). According to whether embryos grow to the blastocyst stage, the samples were divided into two groups, Yes and No, such as the abscissa. The ordinate of the ordinate represents the ratio of mt/gDNA in the culture solution. One black dot or black square represents one sample. Data are means ± SEM.
4. Blastulation time (D5/D6): A total of 44 (51.7%) embryos developed to blastocyst stage on D5, and 41 (48.3%) embryos developed to blastocyst stage on D6. In D3 culture medium, the average numbers of mt/gDNA ratio of the embryos developed to blastocyst stage on the D5 and D6 were 148.5 (SEM = 26.61) and 112.13 (SEM = 23.04) respectively with no significant difference (P = 0.27). In D5 culture medium, the average numbers of mt/gDNA ratios on D5 and D6 were 81.43 (SEM = 25.46) and 82.63 (SEM = 29.37) respectively with no significant difference (P = 0.61) (Fig. 7).

**Figure 7:**
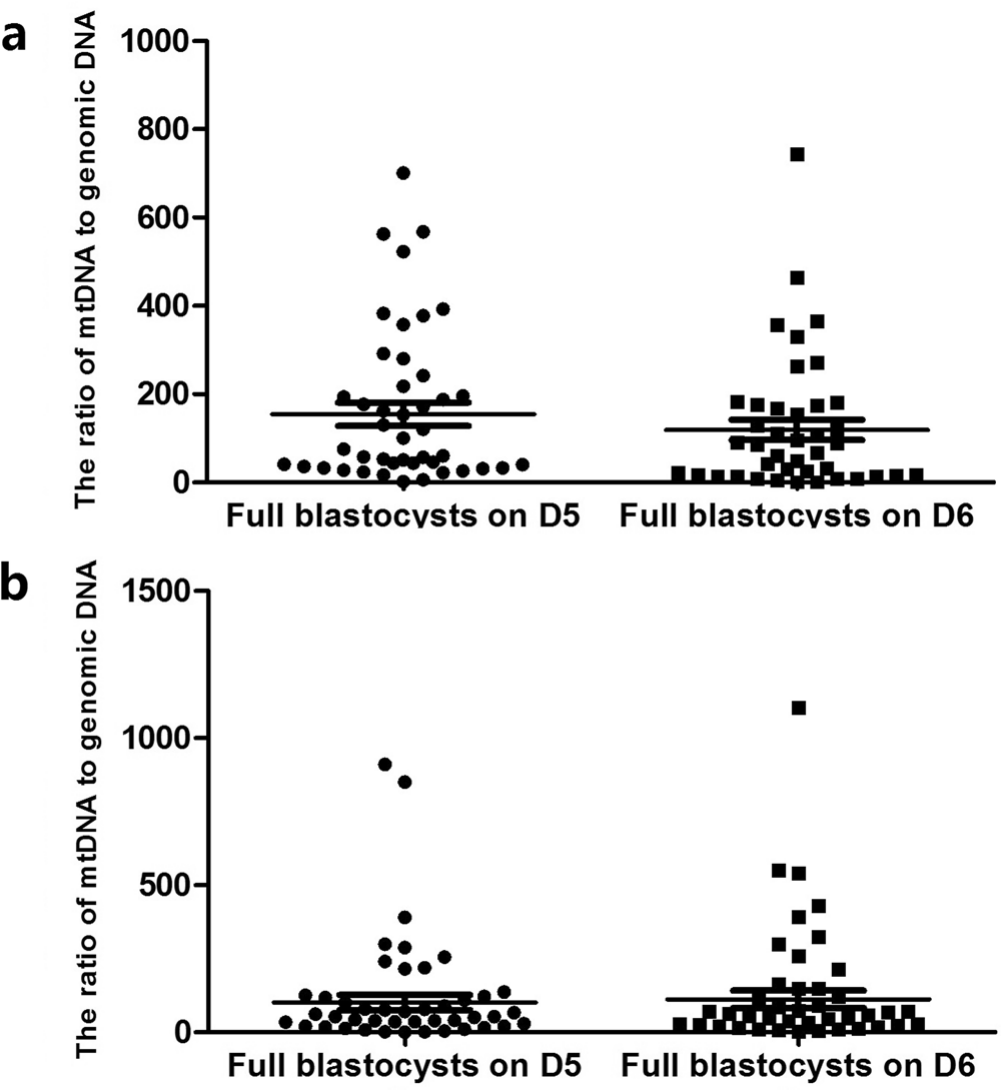
Mt/gDNA ratio in embryo culture medium corresponding to different time of blastulation. At D3, the mt/gDNA ratio corresponding to different time of blastulation in the embryo culture medium (**a**). Mt/gDNA ratio corresponding to different time of blastulation in D5 embryo culture medium (**b**). According to the time when embryos grow to the blastocyst stage, samples were divided into two groups, D5 and D6, such as the abscissa. The ordinate represents the ratio of mt/gDNA in the culture broth. One black dot or black square represents one sample. Data are means ± SEM.

### Age of women and mt/gDNA ratio

**Figure 8:**
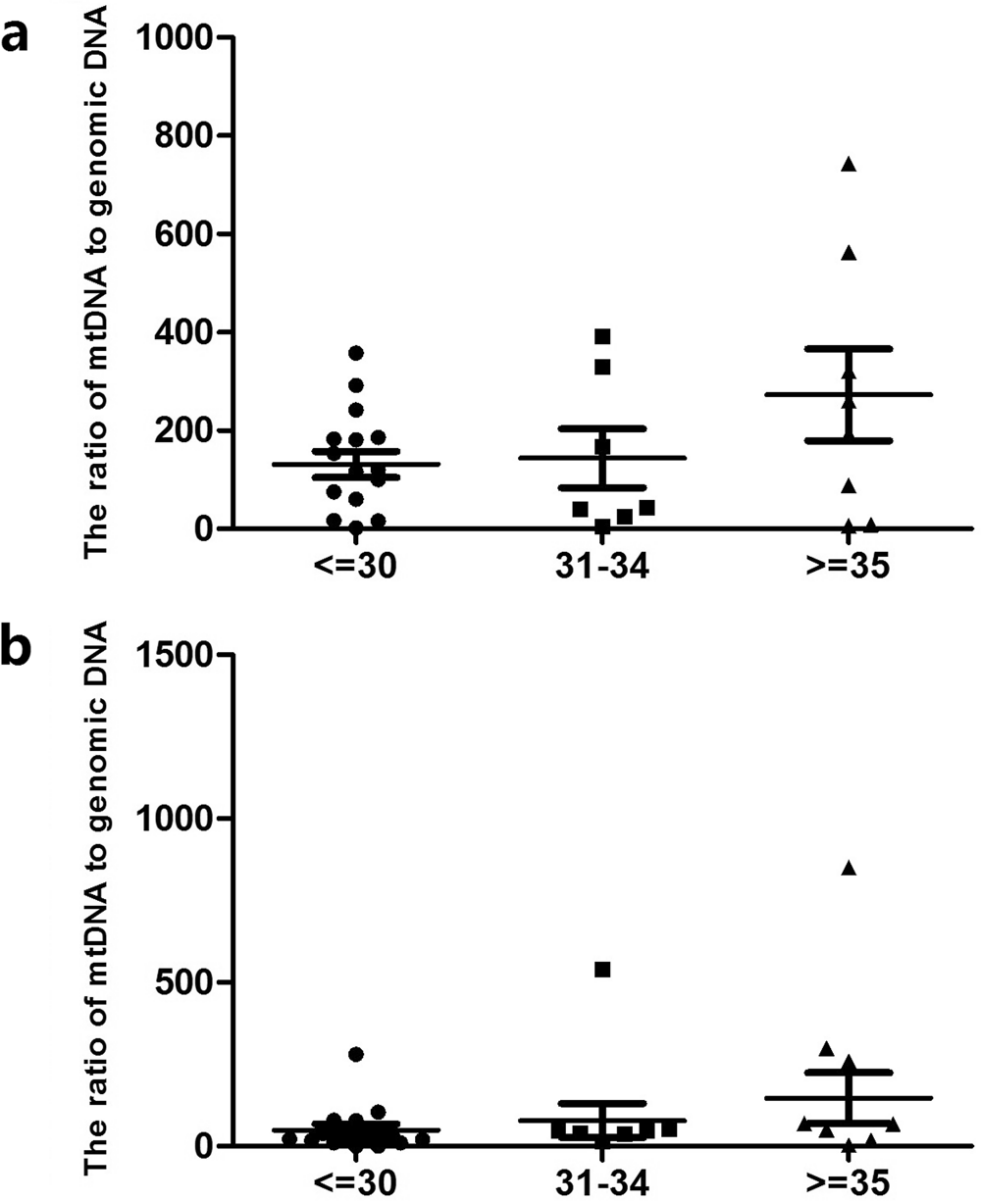
Mt/gDNA ratio in embryo culture medium corresponding to different age of woman (regardless of euploidy). At D3, the mt/gDNA ratio in the embryo culture medium corresponding to different age of woman (**a**). Mt/gDNA ratio corresponding to different time of blastulation in D5 embryo culture medium (**b**). According to the patient’s age, they were divided into three groups, which are less than or equal to 30 years old, greater than 31 years old and less than 34 years old and greater than or equal to 35 years old (<=30, 31–34 and >=35), such as the abscissa. The ordinate represents the ratio of mt/gDNA in the culture medium. Data are means ± SEM.

According to age, the patients were divided into three groups of <=30 years old (A), 31–34 years old (B), and >=35 years old (C). There were 8 cases and 15 embryos in group A, 5 cases and 7 embryos in group B, 6 cases and 8 embryos in group C. When analyzing euploid embryos only, the average numbers of D3 mt/gDNA ratios were 136.86 (SEM = 26.48), 161.17 (SEM = 59.95) and 267.5 (SEM = 93.48), respectively with no significant difference (P_AB_ = 0.82, P_BC_ = 0.47, P_AC_ = 0.40). The average numbers of D5 mt/gDNA were 53.21 (SEM = 18.21), 70.83 (SEM = 51.99) and 109.43 (SEM = 77.23), respectively with no significant difference (P_AB_ = 0.60,P_BC_ = 0.18,P_AC_ = 0.24) (Fig. 8).

### Relationship between mt/gDNA ratio and pregnancy outcome

**Figure 9:**
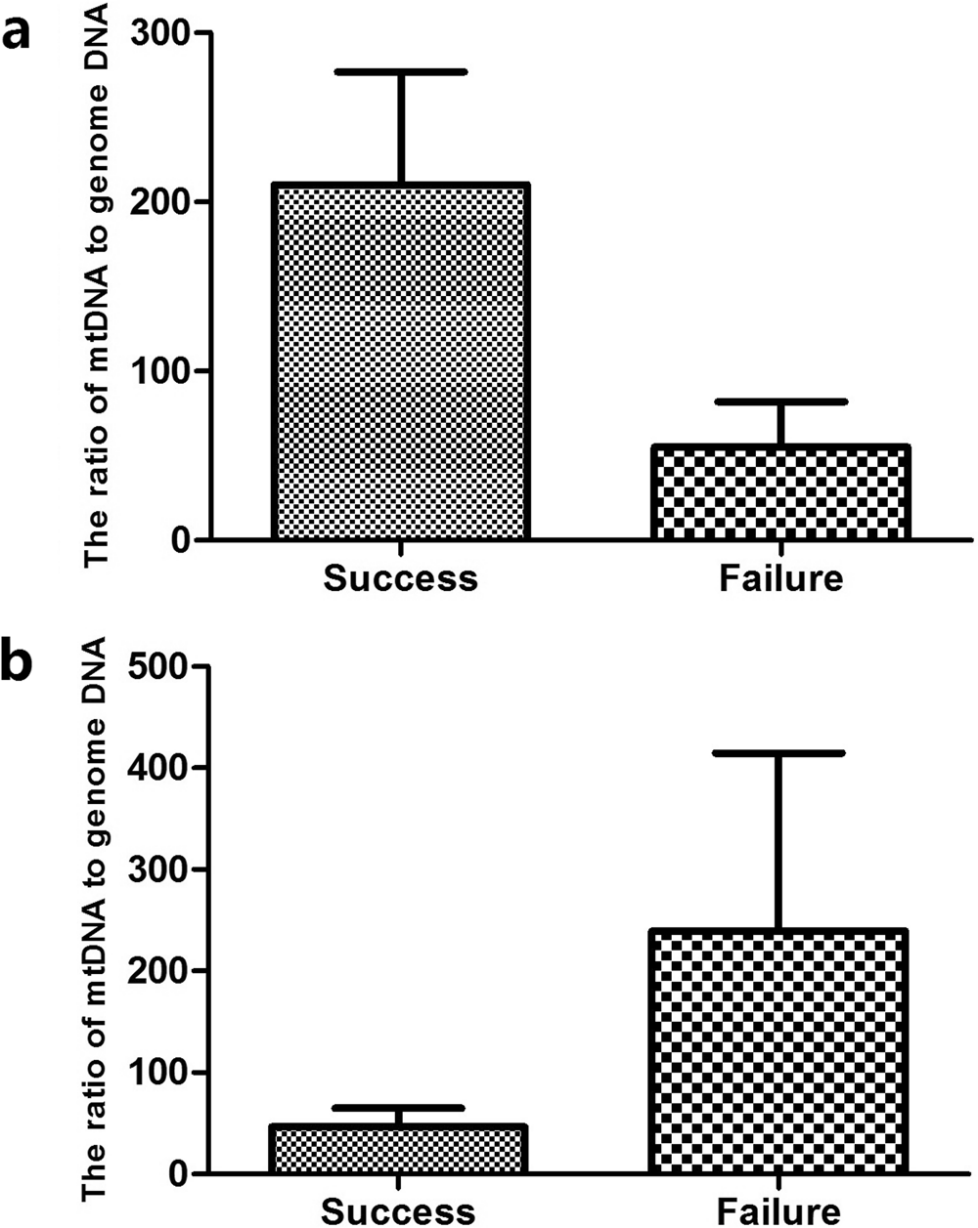
The mt/gDNA ratio for different pregnancy outcomes. At D3, the mt/gDNA ratio in the embryo culture medium corresponding to different pregnancy outcomes (**a**). Mt/gDNA ratio corresponding to different pregnancy outcomes in D5 embryo culture medium (**b**). Based on the pregnancy outcome of the embryo, the samples were divided into two groups, success and failure, such as the abscissa. The 28-day B-ultrasound can monitor the gestational sac and fetal heartbeat as the success criteria. The ordinate of the ordinate represents the ratio of mt/gDNA in the culture medium. Data are means + SEM.

A total of 12 of the 52 cases were transplanted, and one embryo was transplanted in each case (Table S1). And the rest were frozen embryos that had were not transplanted. The 28-day B-ultrasound gestational sac and fetal heart rate were the criteria for success. Of the transplanted cases, 6 were successful and 6 failed. The results showed that on D3, the average numbers of mt/gDNA corresponding to pregnancy success and failure were 210 (SEM = 66.86) and 55.17 (SEM = 26.68) (P = 0.089). On D5, the average numbers of mt/gDNA corresponding to the success and failure of pregnancy were 46.33 (SEM = 18.65) and 239.17 (SEM = 175.7), (P = 0.37) (Fig. 9).

## Discussion

In this study, culture medium of the embryo on D3 and D5 before freezing (transplantation) was collected, and the mt/gDNA in the culture medium was detected using NGS to examine changes of released DNA during the culture process. We believe that the observed low baseline levels of DNA were derived from protein supplements originally contained in the culture medium. The main protein supplement was human serum albumin, which had a primary binding affinity of DNA^26,27^. When the commercial medium and protein supplements were analyzed separately, the highest level of DNA was found in 100% serum replacement supplement^28^. In our study, there was no significant difference in mt/gDNA ratio between NC1 and NC2, indicating that human operation and culture environment did not significantly affect the DNA content in the culture medium. Results showed that there was no significant correlation between euploidy and mt/gDNA of the embryo, but the average number of D3 and D5 in the normal group was lower than that in the abnormal group. There was a certain association between the two, but mt/gDNA could not be used as a clinical indicator to predict the ploidy of embryonic chromosomes as the changes were not statistically significant.

Based on our results, the ratio of mt/gDNA in D3 and D5 was increased slightly with the decrease of embryonic ICM score, but there was no statistical difference. Similarly, as the TE score decreased, the mt/gDNA in D3 and D5 also showed a small increase, and there was no statistical difference either. In addition, the proportion of fragments contained in the blastocyst did not significantly affect the mt/gDNA ratio in the medium (D3 and D5). Therefore, it appeared that mt/gDNA was related to the quality of embryos to a certain extent, but it could not be used as a clinical indicator in predicting the formation of capsules and superior embryos.

From D3 to D5, the mt/gDNA of embryos with successful pregnancy showed a downward trend, while the trend of non-pregnancy showed an upward trend, which was consistent with the trend of forming blastocysts. However, the changes did not reach the level of statistical difference. It could be speculated that mt/gDNA might have a certian correlation with embryo quality, but it could not be used as a clinical indicator to predict pregnancy outcome.

In recent years, the role of mitochondria in embryonic growth and development has attracted wide attention. There are different options on whether mitochondria can be used as a predictor of embryo quality. Some believed that mitochondrial DNA copy number in culture medium could be used as a non-invasive predictive index^23,24^. However, others believed that mtDNA content had no correlation with blastocyst ploidy, age or viability^25^. The results of our study, however, are inclined to show a relationship between mitochondrial DNA and embryo quality and development, but the relationship was not significant enough to be used as an indicator to judge the quality of the embryos.

The conclusion of our study is different from those of the previous reports for several reasons: (1) Most of the studies that support mtDNA as a clinical predictor were based on the recognition of the “silent embryo hypothesis”. This hypothesis was derived from early research on cattles and believed that there was a “mitochondrial genetic bottleneck”^29–31^. The copy number of mtDNA is significantly increased during oogenesis^12,32^ and is actively reduced during pre-implant development^29^. The mtDNA sequence variants segregate between generations despite the high mtDNA copy number in the oocyte^23^. This led to the concept of “mitochondrial genetic bottleneck”, which limits the process by which mtDNA molecules travel from the mother to the fetus and the offspring. Subsequent studies suggested that the copy number of mtDNA was significantly increased during egg production in large mammals^12,32–36^ and then rapidly decreased during the pre-implantation developed phase^31,37^. However, a similar study on mice reached a different conclusion^33,38,39^. It appeared that similar studies of different species yielded different results. (2) At the same time, the mechanism of the mitochondrial genetic bottleneck mentioned above is still controversial. Some studies suggested that mitosis of mtDNA occured before primordial germ cell expansion to gonadal colonization^40^. On the other hand, other studies suggested that mtDNA bottlenecks occurred in the reduction of mtDNA copy number during development before implantation^39^. We collected the culture medium on D3 and D5 of early embryo development, and the results only reflected the changes of this stage. (3) Concerning target genes and detection technology, previous studies selected D-loop gene or other short fragments in mitochondrial DNA as the detection target to represent the copy number of mitochondrial DNA^35,41^. Studies showed that environmental stress might affect the transient synthesis of mitochondria after fertilization^42^. The D-loop region is more susceptible to variation due to environmental stress than other regions of mitochondrial DNA^43^. At the same time, previous studies used qPCR combined with specific probes to detect the D-loop region of mitochondrial DNA^35^, which increased the possibility of unstable results. Another study chose Alu, a multiple copies gene, as a detection fragment^44^, which was not technically superior to that of a single copy gene, and the frequency and composition of the Alu sequence are significantly different in population^45^. multiple annealing and looping-based amplification cycles (MALBAC) and NGS has been successfully used in various studies of DNA at single cell level, such as detecting single nucleotide variation (SNV) and copy number variation (CNV), analyzing meiotic recombination^46,47^, and performing PGS of *in-vitro* fertilized embryos^47,48^. Studies have also shown that NGS is sensitive enough to detect small differences between subculture populations from the same cell line and that MALBAC-NGS is also suitable to study mtDNA copy number in single cells with good reproducibility^49^. Most of the samples used in previous studies were from multiple clinics^22–24,28,50^, and the samples used in this study were from a single clinic. Factors such as media, culture temperature, biopsy techniques, and equipment used during sample collection would affect the outcome. (4) There were other contradictions among previous reports. In some studies samples were collected from both the cleavage and/or the blastocyst stages^28,50^, while other studies collected samples from the cleavage stage only^23^. The above may provide some but not all explanations for the discrepancy.

We conclude that the number of mitochondrial DNA copies released from embryos into the culture medium is related to embryo quality and development during *in vitro* culture, but it would not be a reliable clinical indicator to evaluate or forecast embryo development.

## Methods

### Patients selection

We recruited 52 couples (median maternal age 32.7 years) from Jinjiang Women and Children’s Hospital, Chengdu, China., who gave written informed consent. All couples underwent intracytoplasmic sperm injection (ICSI). This prevented paternal contamination from sperm and limited the maternal contamination from cumulus cells. Ethical approval was obtained from the Scientific and Ethical Committee of Jinjiang Women and Children Hospital for the experimental protocol (2018 KY-001). All clinical procedures including ova collection, *in vitro* fertilization, embryo culture and implantation were performed according to the standard hospital protocol (CDXN/QD-EMBYO-02-17) that has been approved by the Ethical Committee of Jinjiang Hospital which was accredited by ISO9001 and JCI (Joint Commission on Accreditation of Healthcare Organizations). Patient consent was obtained for each embryo sample used in this study.

### Gamete preparation and ICSI procedure

#### Oocyte preparation

After the follicle became mature, human chorionic gonadotropin (HCG) was injected. Thirty-four to 36 hours later transvaginal ultrasound-mediated oocyte retrieval was performed. The ova were repeatedly pipetted in GMOPs (Ref. No. 10130, Vitrolife, Sweden) containing hyaluronidase (Ref. No. ART-4007-A, SAGE, US) for 10–30 s before moving to GMOPS (Ref. No. 10129, Vitrolife, Sweden) without HSA. When the granulosa cells were removed completely, the ova was transferred into culture medium G-IVF PLUS (Ref. No. 10136, Vitrolife, Sweden) covered with OVOIL (Ref. No. 10029 Vitrolife, Sweden). The culture dish was incubated in an incubator containing 6% CO_2_ in 37 °C for 1–1.5 hours.

#### Sperm preparation

We use the upstream method for semen optimization. Detailed steps are as follows:

1. The sample cup containing the specimen was placed in a 30 ± 0.5 °C electrothermal incubator for liquefaction. Specimens were processed within 60 minutes of sperm extraction.
2. Add 1.5 ML Sperm Rinse (Ref. No. 10101, Vitrolife, Sweden) solution to the centrifuge tube (Ref. No. 352099, BD Falcon, United States).
3. Mix the semen sample thoroughly and carefully add the semen to the 1.5 ML Sperm Rinse, taking care to maintain the liquid interface. The tube was tilted at 45° and incubated at 37 °C for 60 minutes.
4. Gently return the tube to erect and transfer the top 1 mL culture to the centrifuge tube (Ref. No. 352003, BD Falcon, United States). The semen containing the semen was placed in a 30 °C, 5% CO_2_ incubator for later use.

Then sperms were magnified 200 to 400 times under an inverted microscope. According to the protocol recommended by World Health Organization (WHO) criteria (2010), sperm morphology was assessed. Sperms of good morphology were selected and immobilized with the injection pipette.

### ICSI

We used natural cycle ICSIs but did not use FSH for *in vitro* ovum maturation. We only selected naturally occurring MII (second metaphase) cells for intracytoplasmic sperm injection. A total of 585 eggs were taken from 52 cases, of which 512 oocytes were in MII. The number of eggs taken in each case and the number of oocytes reached MII in each case can be found in Supplement Table 2. With polar body at the 12 o’clock position, oocytes were held by the holding pipette and the injection pipette was inserted into the oocyte at the 3 o’clock position.

### Embryo culture

Each ova was cultured in a micro-culture well Embryoscope Culture Dish (Vitrolife, Sweden). There were 25 µl G-1 PLUS (Ref. No. 10128, Vitrolife, Sweden) culture medium containing 5% HSA (Ref. No. 10064, Vitrolife, Sweden) in each well covered with OVOIL (Ref. No. 10029 Vitrolife, Sweden) and. Embryo culture was under an oxygen concentration of 5%. In order to allow the embryo culture to reach a temperature suitable for embryonic growth and development, and to bring the pH of the medium to the optimal pH range required by the instructions, we usually incubate the embryo culture media for 10 h before use. On the third day, the embryos that were developed to 6–8 cells were transferred into 25 ul of G-2 PLUS (Ref. No. 10132, Vitrolife, Sweden) containing 5% HSA and cultured until the 5–6th day. The blastocysts were scored according to the method of Gardner *et al*.^51^. The blastocysts with good scores were selected for transplantation.

### Sample collection

#### Embryo culture medium

The culture fluid was replaced on the third day of embryo culture. At this time, 20 ul of the original culture fluid was collected into a RNase-DNase free PCR tube as a D3 medium sample. At the fifth or sixth day, 20 μl of blastocyst culture fluid corresponding to each blastocyst was collected as the D5 medium sample.

#### TE

The blastocysts were then placed individually in a biopsy dish containing 20 µL of G-MOPS PLUS (Ref. No. 10130, Vitrolife, Sweden) under oil for biopsy. Trophectoderm cells were encouraged to herniate from the zona by applying gentle suction with the biopsy pipette.

#### Negative control (NC)

The culture medium identically processed but did not contain embryo was used as a negative control (NC1) and unused culture medium was also used as a negative control (NC2).

### WGA and NGS

The MALBAC method was applied to WGA according to manufacture’s protocols (catalog no. YK001B; YIKON Genomics). Briefly, amplification begins by annealing DNA to a random primer pool, each with a common 27 nucleotide sequence and 8 variable nucleotides. Prior to amplifying the DNA index to ~2 μg for NGS, a quasi-linear pre-amplification step was performed. Using a Covaris S2 system (Covaris, USA), approximately 400 ng of purified DNA was sonicated into a fragment of approximately 150–250 bp and purified using the Qiagen PCR purification kit (Qiagen, Germany). A sequencing library DNA library preparation was then constructed using NEB Next Ultra (New England Biolabs, UK). The constructed library was sequenced on an Illumina HiSeq2500 platform (Illumina, USA).

### Data analysis

The MALBAC amplified genome of each sample was sequenced using the Illumina HiSeq. 2500 platform for a total of approximately 1.5 million reads. The NGS data was analyzed and the chromosomal copy number variation was investigated as described in previous reports^48,52,53^. The high quality read numbers were counted along the whole genome with a bin size of 1.5~2 Mb and normalized by the GC content and a reference dataset. An increase of 2 to 3 copies of the copy number results in a 50% increase in the reading of the particular genomic fragment, while a decrease in the copy number from 2 to 1 results in a 50% reduction in the genomic fragment reading. Mitochondrial content is quantified in the form of copy number/nuclear genome. Sequencing reads were mapped to the mitochondrial genome and were counted and normalized to autosomes. Mitochondrial copy numbers per nuclear genome (MCN) were calculated with the following equation (1). Independent sample t test was used to assess the relationship between mt/gDNA and different embryo quality indicators.

1 $${\rm{MCN}}=\frac{{\rm{autosome}}\,{\rm{mappable}}\,{\rm{region}}\ast 2\ast {\rm{mitochondr}}\,{\rm{ion}}\,{\rm{mapped}}\,{\rm{reads}}}{{\rm{autosome}}\,{\rm{mapped}}\,{\rm{reads}}\ast {\rm{mitochondr}}\,{\rm{ion}}\,{\rm{mappable}}\,{\rm{region}}}$$

### Statistical analyses

The Mann-Whitney test was used to compare the mtDNA/gDNA ratio in spent embryo media and media controls (GraphPad; GraphPad Software). Additionally, it was used to compare differences in mtDNA/gDNA between groups when chromosome euploidy, cystic outcome (means blastulation outcome), and capsular time were different. Independent sample t-test was used to compare differences in mtDNA/gDNA between groups when ICM score, TE score, fragment ratio, and female age were different (SPSS). The independent sample t-test was also used to compare differences in mtDNA/gDNA between the two groups (n = 12) when pregnancy outcomes were different (SPSS). The results were considered statistically significant at P < 0.05.

## Supplementary information

Dataset 1 and Dataset 2
