# Supplementary material for: Mitochondrial DNA and genomic DNA ratio in embryo culture medium is not a reliable predictor for in vitro fertilization outcome
Source: Sci Rep. 2019 Mar 29;9:5378. doi: 10.1038/s41598-019-41801-1 (PMC6441050; doi:10.1038/s41598-019-41801-1)
Supplement: Supplementary file 1 — Dataset 1 and Dataset 2 [file 41598_2019_41801_MOESM1_ESM.doc]

**Mitochondrial DNA and genomic DNA ratio in embryo culture medium is not a reliable predictor for in vitro fertilization outcome**

**Xinyue Zhang**^1^**, Yue Sun**^2^**, Xin Dong**^1^**, Jianming Zhou**^1+^**, Fubo Sun**^1+^**, Tingting Han**^1+^**, Ping Lei**^1+^**, Rurong Mao**^1+^**, Xuzhou Guo**^1+^**, Qi Wang**^1+^**, Penghao Li**^1^**, Ting Qu**^1^**, Jihua Huang**^1^**, Lingxiao Li**^1^**, Tianhua Huang**^1^**, Ying Zhong**^1*^**, and Jiang Gu**1,3,4 *

^1^Jinxin Research Institute for Reproductive Medicine and Genetics, Chengdu Jinjiang Hospital for Maternal and Child Health Care, 66 Jingxiu Road, Chengdu, 610066, China.

^2^Department of Clinical Research, Yikon Genomics Co. Ltd., Building 26, 1698 Wangyuan Road, Fengxian District, Shanghai, 201499, China.

^3^ Laboratory of Molecular Pathology, Center of Molecular Diagnosis and Personalized Medicine, Provincial Key Laboratory of Infectious Diseases and Molecular Pathology, Shantou University Medical College, Shantou, China.

^4^Department of Pathology, Beijing University Health Science Center, Beijing, China.

*Correspondence and requests for materials should be addressed to J.G. (email: 2523381625@qq.com)

Supplement Table 1. Details of embryos biopsied and transplanted and their outcomes.

| Embryo number | Biopsied（Yes/No） | Transferred  （Yes/No） | Pregnant  （Yes/No） |
| --- | --- | --- | --- |
| 1 | Yes | No |  |
| 2 | Yes | No |  |
| 3 | Yes | No |  |
| 4 | Yes | Yes | No |
| 5 | Yes | No |  |
| 6 | Yes | No |  |
| 7 | Yes | No |  |
| 8 | Yes | Yes | Yes |
| 9 | Yes | No |  |
| 10 | Yes | No |  |
| 11 | Yes | No |  |
| 12 | Yes | No |  |
| 13 | Yes | No |  |
| 14 | Yes | No |  |
| 15 | Yes | No |  |
| 16 | Yes | No |  |
| 17 | Yes | No |  |
| 18 | Yes | No |  |
| 19 | Yes | No |  |
| 20 | Yes | No |  |
| 21 | Yes | No |  |
| 22 | Yes | No |  |
| 23 | Yes | Yes | No |
| 24 | Yes | No |  |
| 25 | Yes | No |  |
| 26 | Yes | No |  |
| 27 | Yes | No |  |
| 28 | Yes | No |  |
| 29 | Yes | No |  |
| 30 | Yes | No |  |
| 31 | Yes | No |  |
| 32 | Yes | Yes | No |
| 33 | Yes | No |  |
| 34 | Yes | No |  |
| 35 | Yes | No |  |
| 36 | Yes | No |  |
| 37 | Yes | Yes | Yes |
| 38 | Yes | No |  |
| 39 | Yes | Yes | Yes |
| 40 | Yes | No |  |
| 41 | Yes | No |  |
| 42 | Yes | No |  |
| 43 | Yes | Yes | No |
| 44 | Yes | No |  |
| 45 | Yes | No |  |
| 46 | Yes | No |  |
| 47 | Yes | No |  |
| 48 | Yes | No |  |
| 49 | Yes | No |  |
| 50 | Yes | No |  |
| 51 | Yes | No |  |
| 52 | Yes | No |  |
| 53 | Yes | Yes | No |
| 54 | Yes | No |  |
| 55 | Yes | No |  |
| 56 | Yes | No |  |
| 57 | Yes | No |  |
| 58 | Yes | No |  |
| 59 | Yes | No |  |
| 60 | Yes | No |  |
| 61 | Yes | Yes | Yes |
| 62 | Yes | No |  |
| 63 | Yes | No |  |
| 64 | Yes | No |  |
| 65 | Yes | No |  |
| 66 | Yes | No |  |
| 67 | Yes | No |  |
| 68 | Yes | No |  |
| 69 | Yes | Yes | No |
| 70 | Yes | No |  |
| 71 | Yes | Yes | Yes |
| 72 | Yes | No |  |
| 73 | Yes | Yes | Yes |
| 74 | Yes | No |  |
| 75 | Yes | No |  |
| 76 | Yes | No |  |
| 77 | Yes | No |  |
| 78 | Yes | No |  |
| 79 | Yes | No |  |
| 80 | Yes | No |  |
| 81 | Yes | No |  |
| 82 | Yes | No |  |
| 83 | Yes | No |  |
| 84 | Yes | No |  |
| 85 | Yes | No |  |
| 86 | Yes | No |  |
| 87 | Yes | No |  |
| 88 | Yes | No |  |
| 89 | Yes | No |  |
| 90 | Yes | No |  |
| 91 | Yes | No |  |
| 92 | Yes | No |  |
| 93 | Yes | No |  |

A total of 93 embryos were biopsied, among which 12 were transplanted. Half resulted of in successful pregnancy. Details are shown in the table.

Supplement Table 2. Number of oocytes taken from each patient and number that reach the second stage of meiosis (MII) and received ICSI.

| Sequence number of patient | Number of eggs taken | Number of second metaphase |  | Sequence number of patient | Number of eggs taken | Number of second metaphase |
| --- | --- | --- | --- | --- | --- | --- |
| 1 | 15 | 13 |  | 27 | 24 | 21 |
| 2 | 14 | 13 |  | 28 | 17 | 15 |
| 3 | 4 | 4 |  | 29 | 11 | 10 |
| 4 | 8 | 7 |  | 30 | 10 | 9 |
| 5 | 11 | 9 |  | 31 | 6 | 6 |
| 6 | 12 | 12 |  | 32 | 14 | 13 |
| 7 | 12 | 10 |  | 33 | 10 | 9 |
| 8 | 18 | 13 |  | 34 | 6 | 6 |
| 9 | 13 | 12 |  | 35 | 15 | 11 |
| 10 | 14 | 14 |  | 36 | 18 | 13 |
| 11 | 14 | 11 |  | 37 | 11 | 11 |
| 12 | 16 | 12 |  | 38 | 5 | 4 |
| 13 | 18 | 15 |  | 39 | 15 | 15 |
| 14 | 5 | 5 |  | 40 | 11 | 11 |
| 15 | 2 | 2 |  | 41 | 5 | 5 |
| 16 | 5 | 3 |  | 42 | 4 | 4 |
| 17 | 5 | 4 |  | 43 | 10 | 6 |
| 18 | 13 | 8 |  | 44 | 6 | 6 |
| 19 | 3 | 2 |  | 45 | 5 | 5 |
| 20 | 4 | 4 |  | 46 | 10 | 9 |
| 21 | 7 | 6 |  | 47 | 9 | 7 |
| 22 | 6 | 5 |  | 48 | 3 | 3 |
| 23 | 23 | 22 |  | 49 | 3 | 3 |
| 24 | 30 | 28 |  | 50 | 15 | 13 |
| 25 | 11 | 10 |  | 51 | 16 | 14 |
| 26 | 20 | 19 |  | 52 | 7 | 5 |

A total of 585 eggs were collected from 52 patients. The number of oocytes from each patient and that reached the MII stage and received ICSI are shown in the table.
